# Supplementary material for: Antioxidant Activities, Anticancer Activity, Physico-Chemistry Characteristics, and Acute Toxicity of Alginate/Lignin Polymer
Source: Molecules. 2023 Jul 3;28(13):5181. doi: 10.3390/molecules28135181 (PMC10343217; doi:10.3390/molecules28135181)
Supplement: Supplementary file 1 [file molecules-28-05181-s001.zip › molecules-2424230-supplementary.pdf]

Supplementary Material

Alginate/lignin: Antioxidant Activities, Anticancer Activity, Physico-chemistry Characteristics, and Acute Toxicity

Nguyen Xuan Hoan [1], Le Thi Hong Anh [2], Hoang Thai Ha [2,*], and Dang Xuan Cuong [3]

[1] Faculty of Biology and Environment, Ho Chi Minh City University of Industry and Trade, 140 Le Trong Tan, Tan Phu District, Ho Chi Minh 70000, Vietnam; hoannx@hufi.edu.vn

[2] Faculty of Food Technology, Ho Chi Minh City University of Industry and Trade, 140 Le Trong Tan, Tan Phu District, Ho Chi Minh 70000, Vietnam; anhlth@hufi.edu.vn (L.T.H.A.); haht@hufi.edu.vn (H.T.H.)

[3] Innovation and Entrepreneurship Center, Ho Chi Minh City University of Industry and Trade, 140 Le Trong Tan, Tan Phu District, Ho Chi Minh 70000, Vietnam; cuongdx@hufi.edu.vn

* Correspondence: haht@hufi.edu.vn; Tel.: +84-932-082-199

Abstract: Alginate/lignin is a synthetic polymer rich in the biological activity and is of great interest. Alginate is extracted from seaweed and lignin is extracted from corn stalks and leaves. Antioxidant activities of alginate/lignin were evaluated such as total antioxidant activity, reducing power activity, DPPH free radical scavenging activity, and α – glucosidase inhibition activity. Anticancer activity was evaluated in three cell lines (Hep G2, MCF-7, and NCI H460) and fibroblast. Physico-chemistry characteristics of alginate/lignin were determined through FTIR, DSC, SEM_EDS, SEM_EDS mapping, XRD, XRF, and ^{1}H-NMR. The acute toxicity of alginate/lignin was studied on *Swiss albino* mice. The results demonstrated that alginate/lignin possessed antioxidant activity such as the total antioxidant activity, and reducing power activity, especially, the α – glucosidase inhibition activity, and had no free radical scavenging activity. Alginate/lignin was not be typical in cancer cell lines. Alginate/lignin existed in a thermally stable and regular spherical shape in the investigated thermal region. Six metals, three non-metals, and nineteen oxides were detected in alginate/lignin. Some specific functional groups of alginate and lignin did not exist in alginate/lignin crystal. Elements such as C, O, Na, and S were popular in the alginate/lignin structure. LD_0 and LD_{100} of alginate/lignin in mice were 3.91 g/kg and 9.77 g/kg, respectively. Alginate/lignin has potential for applications in pharmaceutical materials, functional foods, and supporting diabetes treatment.

Keywords: antioxidant; anticancer; glucosidase, physic-chemistry characteristics, acute toxicity

Table S1. Mass and atom of elements in alginate/lignin on SEM_EDS mapping.

Display name	Standard data	Quantification method	Result Type
Map_007_wholespectrum	Standardless	ZAF	Metal

Element	Line	Mass%	Atom%
C	K	22.16±0.08	31.20±0.11
O	K	41.55±0.13	43.91±0.13
Na	K	27.62±0.12	20.32±0.09
S	K	8.66±0.08	4.57±0.04
Total		100.00	100.00
Map_007_wholespectrum			Fitting ratio 0.0210

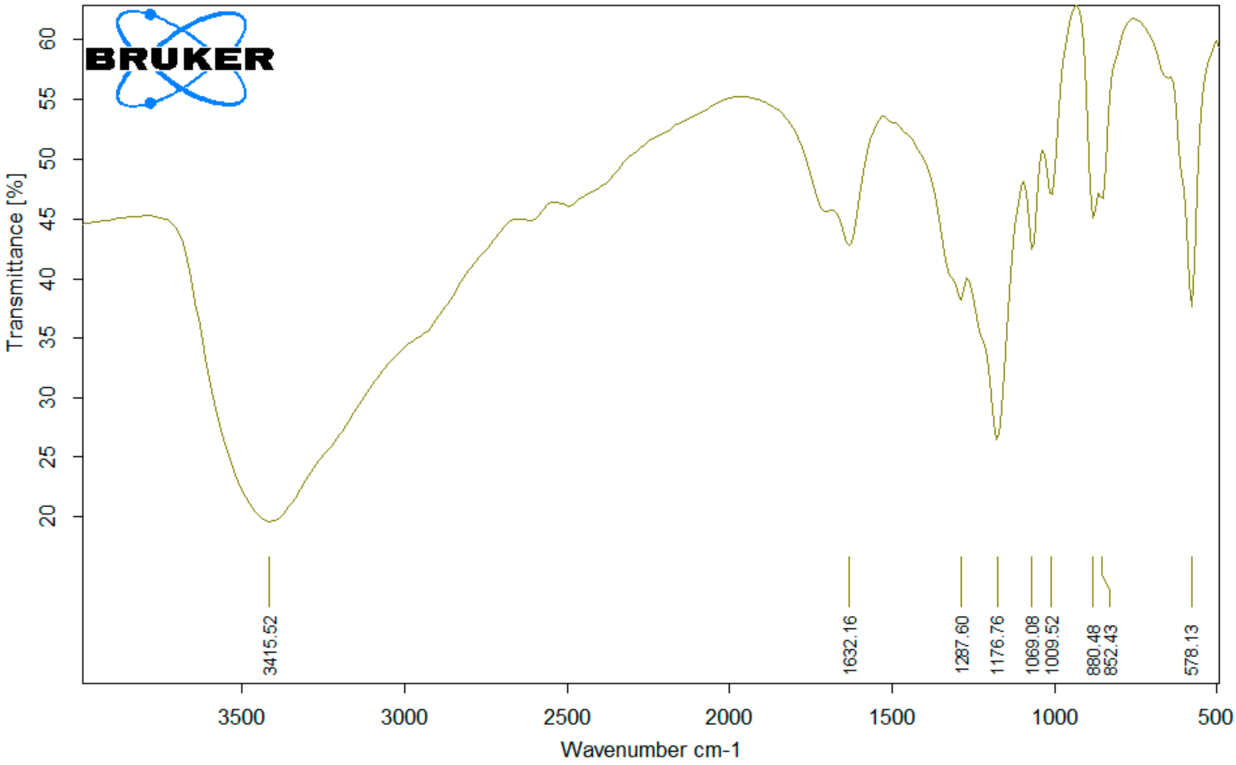

Figure S1. FTIR spectrum of lignin extracting from maize by-products by NaOH solvent.

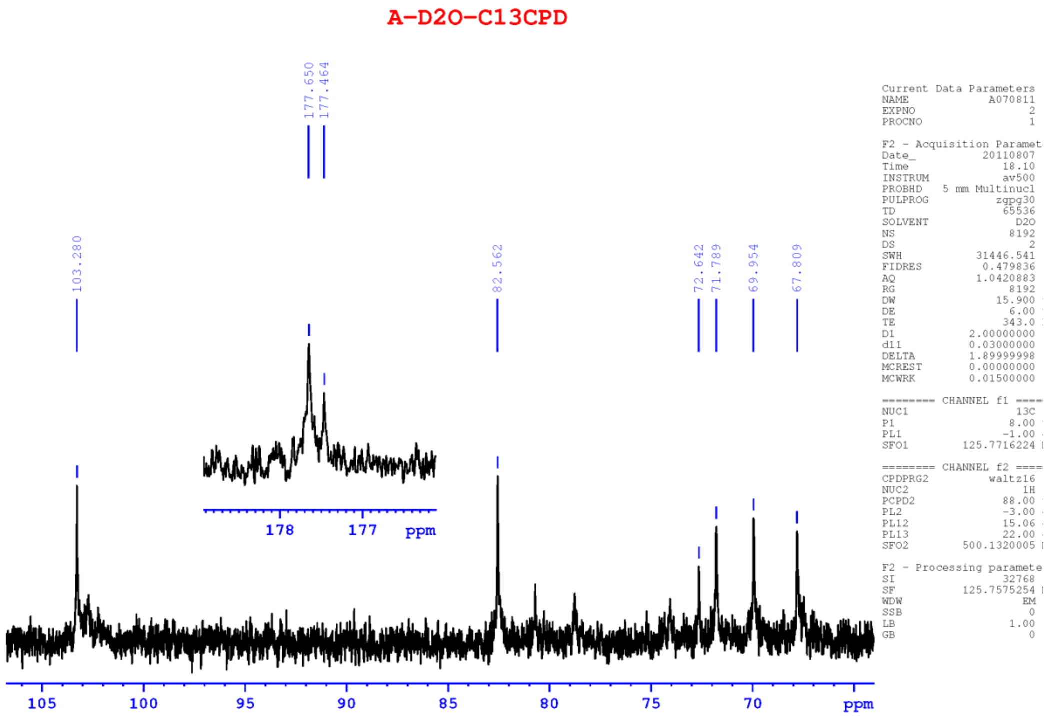

Figure S2. ^{13}C NMR spectrum of alginate extracted from *Sargassum polycystum* [10]

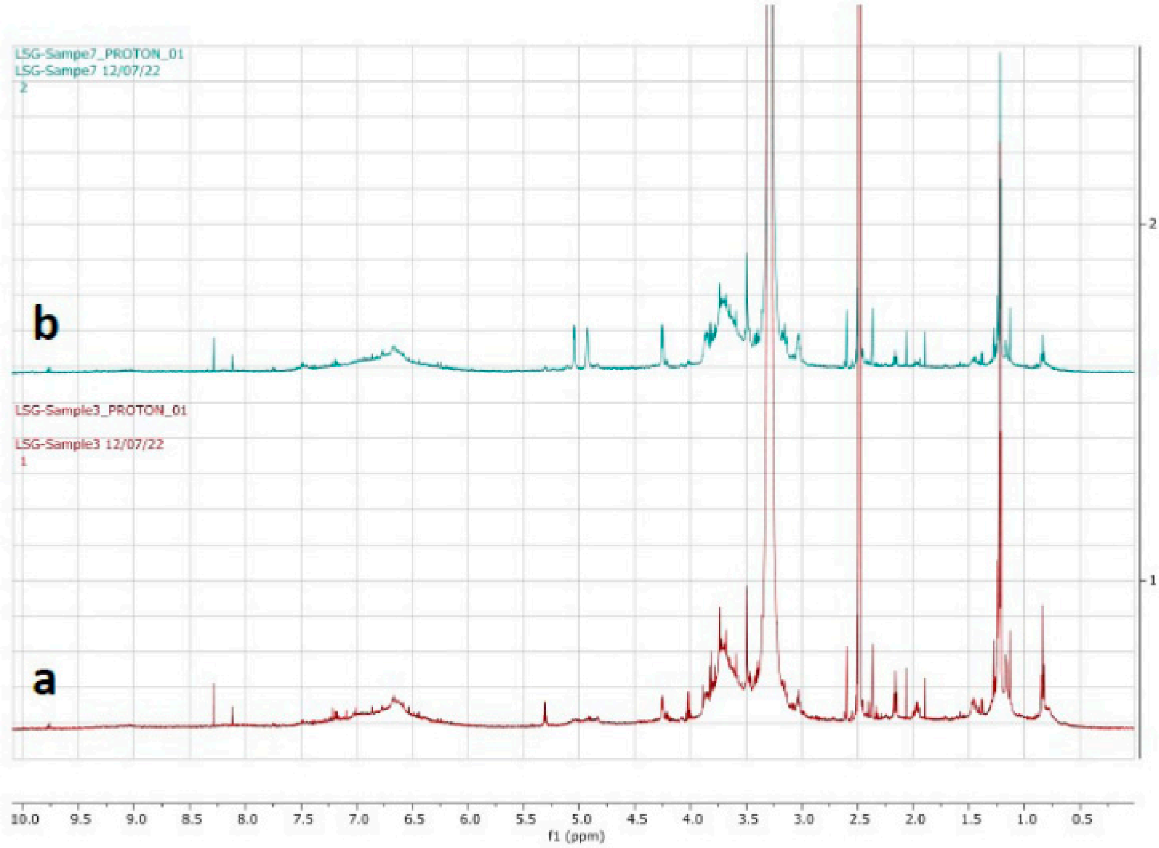

Figure S3. ^{1}H NMR spectra (in DMSO) of lignin extracting from: (a) maize stalks; (b) sugarcane baggase [44]
